# Body Awareness: a phenomenological inquiry into the common ground of mind-body therapies

**DOI:** 10.1186/1747-5341-6-6

**Published:** 2011-04-07

**Authors:** Wolf E Mehling, Judith Wrubel, Jennifer J Daubenmier, Cynthia J Price, Catherine E Kerr, Theresa Silow, Viranjini Gopisetty, Anita L Stewart

**Affiliations:** 1 University of California, San Francisco, Osher Center for Integrative Medicine, California, USA; 2 University of Washington, Department of Biobehavioral Nursing and Health Systems, Seattle, Washington, USA; 3 Harvard University, Osher Research Center, Cambridge, Massachusetts, USA; 4 John F. Kennedy University, Somatic Psychology Program, California, USA; 5 University of California, San Francisco, Department of Social and Behavioral Sciences, California, USA

## Abstract

Enhancing body awareness has been described as a key element or a mechanism of action for therapeutic approaches often categorized as mind-body approaches, such as yoga, TaiChi, Body-Oriented Psychotherapy, Body Awareness Therapy, mindfulness based therapies/meditation, Feldenkrais, Alexander Method, Breath Therapy and others with reported benefits for a variety of health conditions. To better understand the conceptualization of body awareness in mind-body therapies, leading practitioners and teaching faculty of these approaches were invited as well as their patients to participate in focus groups. The qualitative analysis of these focus groups with representative practitioners of body awareness practices, and the perspectives of their patients, elucidated the common ground of their understanding of body awareness. For them body awareness is an inseparable aspect of embodied self awareness realized in action and interaction with the environment and world. It is the awareness of embodiment as an innate tendency of our organism for emergent self-organization and wholeness. The process that patients undergo in these therapies was seen as a progression towards greater unity between body and self, very similar to the conceptualization of embodiment as dialectic of body and self described by some philosophers as being experienced in distinct developmental levels.

## Introduction

A variety of therapeutic approaches often categorized as mind-body approaches claim to enhance body awareness[1] including yoga[2,3], TaiChi, Body-Oriented Psychotherapy[4], mindfulness based therapies/meditation[5], Feldenkrais[6], Alexander Method[7], Breath Therapy[8], and even massage[4,9,10] and mental training for athletic exercise and sport performance[11-13]. These approaches enjoy a growing popularity in the Western world[14]. Enhancing body awareness may not be the main objective for all of these approaches, but it has been described as a key element or a mechanism of action by which they may provide health benefits. Related therapeutic approaches offered by physical therapists in Sweden, Norway and the Netherlands explicitly carry names such as Body Awareness Therapy (BAT) or Body Awareness Program (BAP)[15,16]. These approaches aim to cultivate a particular quality of body awareness characterized by non-judgmental 'mindfulness', "a quality of non-elaborative awareness to current experience and a quality of relating to one's experience with an orientation of curiosity, experiential openness, and acceptance"[17] They have been studied to a preliminary degree for their effects in patients with a variety of medical conditions including chronic low back pain[18-22], pelvic pain[23,24], fibromyalgia[25-27], musculoskeletal pain[28,29], chronic pain in general[29,30], disordered eating and obesity[3,31,32], irritable bowel syndrome[33], sexual abuse trauma[4,34], coronary artery disease[35,36], congestive heart failure[37], chronic renal failure[38], falls in the elderly[39], anxiety[40-42] and depression[43].

Body awareness involves an attentional focus on and awareness of internal body sensations. Body awareness, as we define it here, is the subjective, phenomenological aspect of proprioception and interoception that enters conscious awareness, and is modifiable by mental processes including attention, interpretation, appraisal, beliefs, memories, conditioning, attitudes and affect. We are primarily concerned with those aspects of inner body awareness that, although interacting with thoughts and exteroceptive stimuli, are distinguishable from these and are potentially of key relevance for a deeper understanding of the interaction of mind and body. Therefore, we have restricted our definition of body awareness to the core-awareness of sensations from inside the body and exclude exteroceptive channels. A detailed summary of the current literature and understanding of this ambiguous multi-dimensional construct has been published previously[44].

Mehling et al. suggested that body awareness is a complex, multi-dimensional construct in need of more nuanced conceptualization[44]. In the medical and psychological literature, definitions of body awareness have traditionally been dominated by the concern that heightened body awareness necessarily leads to somatosensory amplification, worsens symptoms of anxiety and hypochondriasis, and is maladaptive for clinical outcomes, such as pain. The term has been used in studies of anxiety and panic disorders to describe a cognitive attitude characterized by an exaggerated patient focus on physical symptoms, magnification ("somatosensory amplification"), rumination, and beliefs of catastrophic out-comes[45]. However, when body awareness is defined as the ability to recognize subtle body cues[37], findings from numerous studies seem to contradict the traditional understanding of body awareness and suggest that it may be useful in the management of chronic diseases such as chronic low back pain[18,46], congestive heart failure[37], chronic renal failure[38], and irritable bowel syndrome[47].

To better understand the conceptualization of body awareness in mind-body therapies that claim to provide benefits from enhancing body awareness, we invited leading practitioners and teaching faculty of these approaches as well as their patients and conducted focus groups. These focus group sessions were conducted as one step in the systematic development of a new multi-dimensional self-report measure of body awareness. The contributions of these focus groups to the measure development and the definition of dimensions within the construct will be discussed in a later paper. The aim of this paper is to articulate the place and function of body awareness in each of the approaches and how these lead to desired outcomes as narrated by practitioners and patients and, thereby, to gain insights into the concept of body awareness as it is understood theoretically, as it is conveyed in practice, and as it is experienced.

## Methods

Fourteen practitioners were approached by written invitation in order to bring eight practitioners together without scheduling conflicts. Practitioners were senior teachers of training institutes with over 10 years of practical experience and living in the San Francisco Bay Area in California, an area exposed to a wide and comprehensive spectrum of mind-body approaches. This group included practitioners who previously had presented their methods in classes for medical students and clinical services at the University of California, San Francisco (UCSF), had participated in research at UCSF or were otherwise nationally and internationally renowned representatives of their respective approaches. The practitioners represented the most common approaches claiming to enhance body awareness[44] and were recruited to participate in two focus groups for two hours each. This paper pertains only to the first focus group session and the discussion of the body awareness construct. The second session had the goal of item collection for the development of a new questionnaire and will be presented in a separate publication. The focus group session was facilitated by an independent, experienced moderator with extensive experience in qualitative studies conducted by universities and major private corporations. The facilitator followed a topic guide developed by the authors.

Each practitioner provided a list of 4-6 clients from their practice. No specific criteria were given for this selection other than representing the variety of their clientele. From this list eight patients were selected by the research team based on a review of the patients' anonymized demographic and clinical background information. We used three criteria in order to ensure a diverse and representative group of participants: (a) Seeking the practitioner for a disease versus other reasons; (b) highest level of education; (c) current employment status. Patients also were recruited to attend two focus groups, however, only the first group for the discussion of the construct is described in this paper. The patient focus group session was facilitated by the same independent moderator following an approved topic guide.

The study was approved by the Committee of Human Research at UCSF. All participants signed informed consent which included the publication of their statements. Participants received the interview questions by mail several days before the focus group and were encouraged to review a 2-page handout explaining various aspects and dimensions of body awareness that might be of importance to their experience. The focus group sessions were conducted between September and December 2009 in a group room at the Osher Center for Integrative Medicine, UCSF, in San Francisco. In addition, practitioners received $100 and patients $50 for the session they participated in.

Practitioners were asked the following questions:

1) "In a nutshell (1 minute), can you tell us about your work and describe a typical client for your method, the reason he/she came to see you, what he/she hopes to get from your method, and what you hope the client gets from it?"

2) "Understanding that different practices use different types of language to describe what they do, what does body awareness mean to you and *how *is it a part of your work?"

3) "If you consider your specific approach, *how*, or *in which way*, do you think it improves a client's body awareness?"

4) "If you talk about body awareness with your clients, how do you talk about it? What words or terms do you use? If you don't verbalize about body awareness, how do you address it in your practice?"

5) "Do you think a client's body awareness has an impact on her/his health condition? Can you please illustrate this for us with an example from your practice?"

Patients were asked the following questions:

1) "Please, tell us in a nutshell (about 1 minute) about the therapy you received and why you went to this practitioner? What was your goal in seeing this practitioner?"

2) "Please, tell us about your experience and what you learned. Did this approach help you pay attention to your body sensations?"

Probing questions: "Do you feel that paying attention to body sensations was helpful, unhelpful, or not relevant to your particular therapy? Did your way of relating to your physical body change as a result of this therapy? How did it change? Did you feel different within your body after this therapy? In which way did you feel different?"

3) "If you saw your practitioner for a specific health condition, do you think this therapy had an impact on your health condition? Can you please illustrate this for us with an example from your experience? Do you think the changes in your body awareness had anything to do with the changes in your health condition?"

All focus group sessions were digitally recorded and transcribed verbatim. Transcripts were reviewed for accuracy by core research team members who witnessed the sessions and took notes. The qualitative team (WEM, JW, VG) used a team-based approach[48] to identify and code themes[49] separately for practitioners and patients following the strategy of Lincoln and Guba[50,51]. The team members read the group discourse with individual accounts closely several times, noting themes and marking sections of text relevant to each theme. The team met regularly to review and compare their identified themes until an agreed upon set of themes for the two groups was developed. Each theme was given a code and one team member (JW) assigned the codes to relevant sections of discourse using ATLAS.ti software. The other two team members read the coded transcripts for final verification. The qualitative team then grouped the themes according to how they addressed the question of interest regarding body awareness. The themes are described and illustrated in the Findings section (Table 1).

**Table 1:** Overview of Themes and Sub-Themes Presented by Practitioners and Patients

Practitioners	Patients
1) Theoretical Stance	1) Reasons for Therapy
A) Integrity of Self	A) specific, individual reason based on symptoms, illness, or sense of dis-ease
B) Human Capacity for Embodiment	B) motivational drive to seek new resources for active coping
C) Central Role Of Embodiment And Integrity Of Self To The Practice	
2) The Practice	2) Engagement in the practice and what happens from the patients' point of view
A) Breath and Breathing	A) Process
B) Repetition and Training	B) Shift in Awareness
	a) Negative Emotion
	b) Body Sensation
C) Noticing/Discriminating/Discerning	C) Engagement in
	a) Self Regulation
	b) Self Care
D) Goal of Practice: Integration of Mind, Body and Life Context	D) Integration of Mind, Body and Life Context
	a)Context
	b) Mind-Body Integration

## Findings

Participants in the first focus group were 8 experienced practitioners representing one or several of the following approaches: Yoga (Iyengar), Yoga Therapy (Desikachar), Tai Chi (Yang style), meditation (in the therapeutic form of Mindfulness-Based Stress Reduction, MBSR, Kabat-Zinn), Feldenkrais method, Alexander technique, Breath Therapy (Middendorf), Somatic Experiencing (Levin), Somatic Therapy (Hanna), Hakomi therapy (Kurtz) and massage. Four practitioners were female and four male. Five had international, teaching credentials and three had national teaching credentials. One practitioner was African-American, all others were Caucasian. Eight patients from these various practices participated in the second focus group. They were predominantly female (1 male patient), varied in years of practice (6 months to > 20 years), reasons for seeing their practitioners (chronic pain 4, HIV 1, stress and sleep problems 1, no health reason 1) and level of education (two did not complete college, one completed college and five had a graduate education). One patient was Asian, all others Caucasian. One consented patient had to cancel due to a car break-down, thus reducing the patient focus group to seven patients.

### Practitioners' Views of Body Awareness

Prior to the first meeting, all participants were in agreement that body awareness is a core feature of their approaches and welcomed the invitation. The group interview focused first on questions relating to the role of body awareness in the participating practitioners' various practices. Interestingly, the practitioners initially responded by clarifying basic theoretical tenets about embodiment, making it clear that they all held to the notion that the mind and body are not distinct entities, but integrated and interactive. To begin, we present this theoretical stance, and then proceed to elements common to their practices.

#### Theoretical Stance

With respect to their theoretical stance, the practitioners' accounts spoke to the following commonalities: (1) integrity of self (mind and body not viewed as separate entities), (2) innate human capacity for embodiment and challenges to achieving embodiment.

##### Integrity of Self

The practitioners voiced a concern that the term body awareness may not reflect their view of the person as an embodied being. Embodiment includes an integration of mind and body and a bodily capacity for knowing. They expressed a preference for the term "self awareness" rather than body awareness. Or conversely, when they used the term body awareness, they emphasized that it is meant to include "every level... the physical, the breath, the mind, the personality and the emotions", that the 'body' of body awareness is inseparable from its functions and all other aspects of self awareness. They could talk about body awareness if it was understood as a core aspect of embodied self-awareness.

"...the term body awareness ... perpetuates the split of the mind/body connection. So that's why Feldenkrais himself would never use the word 'body'. It was always self awareness."

Furthermore, the term "Awareness" was not understood as a purely cognitive capacity, but as a capacity of all aspects of the self, physical, mental, and emotional, interacting and informing each other.

"I would say body awareness for me is self awareness also, and I think of it as self awareness in terms of at every level, you know, and how we use it is really looking at how the different levels of the human system overlap and influence each other, the physical, the breath, the mind, the personality and the emotions."

Beyond the integrated self is the larger context in which the person acts, interacts, defines the self and is defined. So the person is not a discrete entity, but an embodied being enmeshed in and interacting with the world. This enmeshment includes involvement in ongoing goals and commitments as well as relational engagement with others.

"And self awareness ... is the awareness of moving, sensing, feeling and thinking that they happen all at the same time. And that is all for action...self awareness and body awareness through action as you're moving through space, as you're in relationship to another person."

##### Human Capacity for Embodiment

Another aspect of these practitioners' theoretical stance is that people from the start of life are endowed with a capacity for embodiment and integration of self. Initially, embodiment is expressed as a capacity that needs to develop.

"We are not fully embodied as we arrive on the planet as beings. Like the faun or the doe come in and they pop out of their moms and they start prancing around, and they're in their bodies. We as human beings are not. So what we learn in time is how to embody."

And, since this innate capacity for embodiment is not dormant, but part of what it is to be a human being, it is evoked by living in the world and acts quasi 'behind the scene' as a natural developmental tendency with intelligence and purposive intentionality.

"We're trying to locate the innate intelligence, whether it's through movement or patterning or whether it's about psychological, physical, there is an innate intelligence in our body-mind that exists that we really want to support the client in knowing."

This theoretical stance is not understood as simply an abstract concept. It is central to the practice itself.

Central Role Of Embodiment And Integrity Of Self To The Practice

These two aspects of the practitioners' theoretical stance, integrity of self and embodiment, find expression in all aspects of their work with patients. The need for these healing practices arises because although people naturally develop into embodied beings, they also get disrupted or even stuck in this development.

"We all tend to arrest the development of the body awareness, the embodying process, early on at some point. And then we have to continue that on."

The tension and suffering that result from the disruption of this embodiment process mobilizes that "innate tendency to embodiment" and leads patients to seek help with body awareness practices. The motivation is often not clear to the patient. Individuals typically seek out mind-body practices for the relief of pain or other bothersome symptoms. But, the practitioners believe that behind this conscious motivation is a pre-cognitively felt need to resume a stalled process of embodiment.

"Why we come is not often why we really come, or think we come. ... people come because they're in pain, whether it's physical, mental, emotional, spiritual pain. ... [The symptom] gets them in the door, but it is not the real story."

"What they hope to get out of it is relief, of course. ... Even if they're coming with severe pain they'll say that their goals is ... greater flexibility or reduce my pain or whatever. But most people will include something about ease of wellbeing or peace of mind in there."

To these practitioners and their therapeutic practice, therefore, the integrity of self and this dynamic of the embodiment process are central. Addressing one aspect of the patient will engage other aspects.

"So, different tools to work at the physical level, the mental level, emotional level. And a big part of it is connection through the breath and connection with movement and breath to make that connection with the mind and eventually quieting the mind to give them greater connection with their self."

"And over time if enough support is provided, if they learn or unlearn appropriately, then they can develop trust in this innate sort of emergence I think that we're also speaking of: something that's not just the conditioning or not just the ego or the personality, however you like to talk about that. But something that's really reflective of the innate tendency of our organism for emergent self-organization and wholeness."

The integrity of self allows different aspects of the person to be in contact with other aspects. In terms of the practice this intra-self contact can phenomenologically appear like a conversation.

"We're trying to emphasize the experience, that people experience the sensations of the movement of breath. In the classes, in the breath and movement classes through simple sequences that represent, and in the hands-on work we call it: the breath dialogue, where we are basically dialoging with the allowed breath, and help them to sense and understand what's happening with their breath. And let *them *say it."

And, as one practitioner describes it, the conversation does not require words.

"So we don't talk, you know. I don't care what people are feeling, you know, basically. What I want them to do is model the form and that leads to the ability to control the attention and direct the movement.... I want them to have total awareness of what's going on in their thoughts, what's going on in their emotions, what's going on in their body, as they are going through this form. And having gone through this process over the last years, I know that there's all the emotions that come up, and all the sensations, and all of that stuff that goes on. But it's not of any concern to me as the teacher. What I'm concerned with is that they model, because I know from the experience that they will go through all of this other stuff."

Only once these theoretical understandings were made clear did the practitioners turn to the concept of body awareness itself and how it manifested in their practice.

#### The Practice

Although the practitioners represented multiple healing modalities, there were basic similarities in their approaches. These included (1) the use or role of breathing, (2) training and repetition, (3) noticing body sensations, discerning and differentiating changes in the body, thoughts and/or emotions, and (4) body-mind integration as the therapeutic goal

##### Breath and Breathing

The role of breath and breathing was thematic among the practitioners. For the Breath Therapist, breath was central to embodiment and to the healing process.

"So, a breath experience can actually be the allowed breath, the breath that comes and goes on its own, which connects to an intelligence, to an inner intelligence, a resource within, that is seeking a balanced state of being. And we're teaching people to connect with that breath and to learn, as they learn to use that breath, to listen and follow that breath in its development, as it develops throughout the body and clears and integrates patterns of resistance."

But other practitioners also saw breath as a central element to deal with in terms of being a bodily aspect that is affected by experience, particularly negative experiences.

"I'm just reminded that ... Peter Levine once said: "if it doesn't affect the breath then it's not trauma". Right? And so breath is one of those key dimensions."

Breath was seen as a central connector or link between body and mind. In this way it can serve as a tool for the practitioner to use with the patient, a tool that patients can learn to use themselves in their work towards re-integration of mind and body.

"I would say really simply, helping to bring awareness to the breath and coordinating gentle movement with the breath helps to bring that connection to the mind, you know, linking the body and the mind through the breath and coming to that place of greater self-awareness at every level, you know, body, breath, mind, emotions."

"I think the promised land is likewise this sense of embodied self. And ... maybe the most succinct definition of that ... is, I think, probably the free movement of breath among all the systems and tissues."

While a number of practitioners described the focus on breath as central and very useful in their practices, it was not the focus of all the practices. As the following account illustrates, because people are integrated beings, it is not absolutely necessary to focus on the breath or work cognitively with it in order to affect the breath.

"I just thought I'd bring a little diversity with you in and around the breath. I believe breath is the basic pattern, but why in Alexander Technique and in somatic therapy we don't focus on the breath. I don't focus on the breath so much at all. I mean I notice it as a therapist, but if there is something that's happening that needs to shift, that will shift, then the breath will come and be allowed to come, as opposed to the other way around, focusing on the breath in order for a shift to come."

##### Repetition and Training

All of the healing approaches have in common that they are practices, and, for the practice to be effective therapeutically, it has to be learned by the patient. This learning requires training and repetition.

"Something particular to mindfulness is the training aspect of it. It's repetition, it's training."

##### Noticing/Discriminating/Discerning

A central skill that patients learn through training and repetition is the ability to notice sensations, thoughts and feelings as they occur in their actual immediacy. What is noticed might be verbalized or not. The point is that the process of noticing and the learning of differentiated noticing were viewed by the practitioners as a path to integration.

"I would say I welcome and invite everything that you notice what's happening, everything is welcome, and describe, observe and describe what's happening. It could be an image, a sensation, a thought, it could be anything. So it's inviting the client to describe, learn to describe very specifically what is happening, to wake them up to that. Tracking and studying that direct experience in the present moment."

"We just do full throttle attention, because the assumption is people aren't paying attention enough. And so, we just want to develop the attention muscle as much as we can. So we have people bring their full attention to this and then to that and then to your breath and then your body."

Other practitioners described this process as discriminating. However, when they articulated what they meant by discrimination, it sounded like a next step after noticing. After noticing the patient is in a position to decipher what they are experiencing.

"What does the sensation feel like, do I like the sensation, how much of my attention goes to the sensation... You're discriminating, you're kind of figuring out what's going on."

Yet others expressed a preference for the term discernment. Again, though, when described, discernment appears to be a third step in the process toward integration.

"I would use more "discernment". You know, so through the practice, you know, then beginning to discern "oh what's this?" "What's the difference?" "Oh my mind is actually different from my emotions." Or the thoughts are different. Trying to differentiate. The ultimate goal of yoga in many ways is being able to really consciously direct your focus or your attention in one chosen direction. Yet at the same time very much being in a sense of an integrated, embodied self, you know, without dissociating."

So through repetition and training in these skills of noticing, differentiating, and discerning, individuals engaged in mind-body practices that involve the body are immersed in a process that leads to embodiment and integration.

"...the instinct in terms of differentiation and embodiment, though, shouldn't be confused with the process, which is: the process is the careful attunement to the moment-to-moment awareness of process.... *So even though embodiment is the key, not-embodiment is the way."*

This last sentence captures the paradox of these practices: re-integration of mind and body involves learning to fine-tune and break down awareness to attend to the nuanced and various components of internal experience.

##### Goal Of The Practice: Integration of Mind, Body and Life Context

There was strong agreement among the practitioners that their practices held the ultimate goal of integration mind and body in the context of daily life. They saw their practice as a process that moved through four dimensions of body awareness, perceived body sensation, attention, attitude, and integration, and is actualized in daily life.

"I like that idea of the continuum, like that's the end point for us. And we want to do that in action. So can you be aware of that, as you're walking, as you're picking up your baby, as you're sitting in your car, as the boss walks by your desk. To that when you're actually involved in the world, because it doesn't matter what we do on our mat -- do we take that out into the world. So that's a big thing for us, of moving through space in the environment and action."

### Patients' Views of Body Awareness

The patients' responses in the focus group in many ways mirrored, expanded on, or gave another perspective on the practitioners' views of body awareness. In this section we describe the patients' views. We begin with the patients' reason for seeking therapy and then present how the patients describe their experience resulting from their engagement in the practice.

#### Patients' Reason for Therapy

While the practitioners spoke in more general terms about the reasons that their patients sought them out, the patients themselves were very clear and specific about their reasons for seeking therapy. The common theme for everyone was that something was not working well for them and they had reached a point where they felt they had to do something. Many had tried multiple other avenues before they took up the practice they describe in the interview.

"So my goal in going to yoga therapy was to manage chronic pain issues that I had due to some degenerative disc disease and arthritis. And specifically just to keep it at bay so it wouldn't get any worse. Because I had been doing physical therapy for quite some time and that was not providing any relief. In fact I was getting worse. So I started yoga therapy ... my goal is really to manage the pain."

The patients did not come to therapy because of their theoretical understanding of or commitment to embodiment and the integrity of the self that the practitioners held. Their reasons for seeking therapy were individual and personal.

#### Engagement in the Practice and What Happens From the Patients' Point of View

The patients described four aspects of what engagement in the practice entailed and what happened to them in the course of participating in the practice. First, like the practitioners, they saw the practice as a process. Second, they described experiencing a shift in their awareness. Third, they articulated how they learned to engage in self regulation and self care. And, fourth, they described their lived experience of an integration of mind and body in the context of their ongoing lives.

##### Process

Like the practitioners, the patients understood the therapy as a process. And also like the practitioners, the patients recognize the process as ongoing, unfolding and never static.

"But one of the ... from all the stuff I've done is the concept of movement, the lack of movement or the use of movement, whether it's the physical, mental or emotional. And how people move through their process."

Patients confirmed the key role of breath awareness for their practice.

"Meditation is all about paying attention. Of course the basic starting point is with the breath, or with the body in general, but especially the breath."

"Paying attention to the body sensations is -- is vital with breath, because you want to feel how the breath moves through your body. And when breath is in certain parts of your body they will loosen up. They can stretch and they will flow. If you breathe correctly when you move you will actually have more strength and less strain and a lot more ease in your body."

##### Shift In Awareness

Patients described a shift in awareness as a result of the process of engaging in the practice. Although this could be generally described as "body awareness," the patients discriminated between two aspects of this awareness. First, they recounted a shift in awareness of their experience of and response to negative emotion. Second, they described a shift in awareness of bodily sensations.

*Negative Emotion*

The topic of negative emotion, and awareness of a change in the experience, or awareness of emotion came up frequently among the participants. They discussed negative emotional reactions they had prior to engagement in body awareness practices. These previous emotional reactions could be in the form of catastrophizing over pain; alternatively, they could be a more general reaction to a stressful situation.

"On one side you have the *emotional *schemas that you practice, that result because this behavior causes you to do such-and-such. And in the *physical *side, your foot hurts and therefore you do this or you fixate on this; or you think you've got cancer of the foot, or whatever. And that's the same schema. And so we were learning that none of that really holds true, that the schemas are what you get rid of."

"The whole notion of sort of avoiding and pushing away unpleasant experiences that I would just do anything to, you know, avoid like a conflict situation. I'm much more comfortable with that now... Now I can just pay attention to what's happening in the moment and it's much more pleasant to get through it. And the recovery from an unpleasant experience is much quicker as well."

While the practitioners were articulate in talking theoretically about mind-body issues, the patients were articulate in speaking of their lived experience. They described how a mind-body practice altered an emotional reaction or changed the way they responded to emotion.

"How I feel about sensation in my body is different. It used to cause some panic in me if there was any discomfort. And I don't immediately jump to the conclusion that I have terminal cancer if my knee hurts.... So it's nice to have a little bit of space between the sensation and the emotional sort of reaction to it."

In sum, the patients described both a pre-existing relationship between body sensations and emotions and the ways in which that relationship and awareness had changed with their mind-body practice. The practice gave them more possibilities beyond their previous automatic responses to sensation.

*Body Sensation*

Patients described a shift in awareness of body sensations as part of the process of engaging in the practice. The shift in awareness of body sensations then could lead to a change in how the patients responded and related to the sensations.

"And then also just being intuitive and listening to your body more: "I want to hike" "I want to do yoga. I want to do this. Well, maybe you shouldn't do that today. Maybe it would be nice to burn off some stress but maybe you should also listen to your body and not..." So I started also doing less instead of doing more all the time. And I equate that to just being aware and paying attention to my body sensations."

"But just a huge learning for me to really pay attention to where was I getting to that edge of pain. And this -- this just changed the way I started to move, in general, all the way through my day."

This shift in awareness recounted by the patients corresponds to the practitioners' description of leading their patients to notice, and to differentiate and discern among their bodily sensations, cognitions, and emotions.

A key element that changed in their relationship to their body sensations appeared to be the awareness of the differences a) between thinking about a sensation and directly sensing the sensation, and b) between a willful attention and a more relaxed, accepting and allowing attitude in their attention towards these sensations.

"it's the thinking that used to get me all tied up and worked up and tense. But now I can just pay attention to what's happening in the moment and it's much, much more pleasant to get through it. And -- and the recovery from an unpleasant experience is much quicker as well."

"It's like more integration of a releasing of that thought as well as "okay, am I thinking? Is it my emotions? Is it pain?" But how about just like existing in that feeling? .....Here you're still judging and thinking ... and doing ... rather than just: it's there."

"I've just been less judgmental of myself, being more aware of my thoughts and what I attach, what I think and assign to everything. So I've seen a decrease in my judgment and analyzing of situations and just sort of letting them be. And that's definitely helped me: it decreases my stress, it helps me be more open to different possibilities and solutions."

##### Engagement In Self Regulation And Self Care

Learning to notice, differentiate, and discern as described above appears to lead to and to facilitate development of the skills of self regulation and self care.

**Self Regulation**

Self regulation is a skill the patients learned in their practice, which they then applied while doing the practice or when dealing with bodily sensations such as pain. They learned how to accept the limitations of their body and adapt their movements and behavior.

"The awareness of the pain is important so I don't go beyond it. But also learning new techniques of breathing or whatever to adjust."

"I got my first yoga practice ..., very subtle and so for me to retrain and be really aware of where my range of motion was without pushing past it was just a huge learning for me. Just a huge learning for me to really pay attention to where was I getting to that edge of pain. And this just changed the way I started to move, in general, all the way through my day."

Self-Regulation extended to emotion regulation by letting go of judgment and appraisal of life situations.

"The physical moving has done a lot more for helping me be calm when I'm in a stressful situation. So that when the stress arises, you know, when you get to the airport and your plane is delayed nine hours and there's no flights and no hotels and everyone else is sort of screaming, I don't join in that. Now I can just see 'oh I'm feeling a little agitated, time to start breathing.'"

"And realizing that you have the ability to respond rather than react and the degree to which all of us are on auto pilot most of the time. It's like 'no, you have options here, you can choose how to react, how to respond to this situation.'"

And, as some of the patients expressed, this learning process may not involve conscious and purposive mental activities or engage the personal will.

"That in the past if I got the pain I would think: wow, is that an old injury? What did I do before to make all this happen? And in Feldenkrais it was okay, you have some awareness of it and now let it go. And be sure that you don't do any movements that affect that threshold of pain. But to focus on it or analyze it or figure it out or fix it is not the Feldenkrais way."

***Self Care*** is another related skill. It is directed to taking care of the self in life situations that could be challenging. It can involve a change in personal orientation such that self care is a worthy practice rather than something to avoid in subordination to goal accomplishment.

"When I do things I know to what limit I can do now. That if I go beyond two hours of pruning my bushes or something I'm going to have pain somewhere. So I don't do that anymore."

##### Integration of Mind, Body and Life Context

The practitioners described the integration of mind and body in a life context as a goal of their therapies. The patients provided examples of the lived experience of integration within their life contexts.

*Context*

Context is a dimension that the practitioners touched on a number of times and indicated its importance. They indicated that context needed to be included in the construct of body awareness because it is an important part of what is attended to in their practices. The patients elaborated on the issue of context in terms of lived experience in relating to the world and others. Participants described how their relationships with other people also changed as a result of their therapy.

"The more comfortable I become in my body and not into my head the more comfortable I find people are with me, that I relate better and more easily and express myself better and people listen to me more. It's really interesting, because I'm not pushing. I'm just being."

"It's my relationship to my body and my mind and other people. I mean it's amazing how the body awareness has brought awareness through many other things. So this work has helped my relationships with myself and with others."

"A lot of the stuff that you learn in MBSR kind of spills over into real life."

*Mind-Body Integration*

The practitioners held integration of mind-body as the ultimate goal of their therapies: what they hoped their patients would attain. The patients provide an extension of this aim by describing the ways in which they experienced this integration in the course of their ongoing lives.

"It's more than physical! It was, sort-of, an intellectual and emotional response to understand what was appropriate for my body. So I just became much more sensitive to listening to what was going on with my body and the cues that it was giving me."

"As you become more mindful, it's more about just being aware of what is. I may be panicked, but that's neither a good nor a bad thing. It's just a fact of what's happening right at this moment. So having awareness of that is useful for developing mindfulness."

## Discussion

The qualitative analyses of focus groups with representative practitioners of a variety of body awareness practices, and the perspectives of their patients, elucidated the common ground of their joint understanding of body awareness. For them body awareness is an inseparable aspect of embodied self awareness realized in action and interaction with the environment and world. It is the awareness of embodiment as an innate tendency of our organism for emergent self-organization and wholeness. This innate tendency toward embodiment drives the motivation for patients to engage in these practices. Body awareness-enhancing therapies resume an embodiment process that has been disrupted in its unfolding, and these therapies tap into the indivisible integrity of the self, for which also other terms are used, such as the intelligence of the body or an inner resource. Common elements of the represented practices include: the central role of breath awareness for practitioner and/or patient, repetition and training, refinement of noticing, and discriminating and discerning physical sensations. These elements support the common goal of all practices, the integration of mind, body and life context. This process may entail inter- and intra-personal 'conversations' with or without words, cognitive or pre-cognitive. It can be described in terms of shifts in awareness of physical sensations and negative emotions, of engagement in self-regulation, emotion regulation and self care, integration of mind, body and lifeworld context.

The theoretical stance of the practitioners demonstrates a striking parallel to positions presented by phenomenological philosophers who, in the tradition of French phenomenologist Merleau-Ponty[52], attempt to transcend viewing persons in dualistic terms and focus not on "the body" as such but on what it means to be 'embodied'[53,54]. "Embodiment is the human experience of simultaneously having and being a body; the term conceptualizes the body as a dynamic, organic site of meaningful experience rather than as a physical object distinct from the self or mind"[55]. The practitioners of the focus group expressed the absolute need to see body awareness as an inseparable part of self-awareness. The embodied self as the experience of an integration of "all levels: body, mind, breath, emotions, and personality" was viewed as the goal of mind-body approaches, after patients enter these therapies in a less developed, less integrated mode of embodiment.

The process that patients undergo in these therapies was seen as a progression towards greater unity between body and self, very similar to the conceptualization of embodiment as a dialectic of body and self described by some philosophers as being experienced in four levels[56]: 1) in a level labeled "the lived body" the body is taken for granted, and patients are unself-consciously aware or unaware of it, the body often described as "absent"[57]. This state was well-described by the patients when they began their practices. 2) in a level labeled "the objective body state" the body is experienced as opposed to the self. Body and self are in tension with each other or in disunity, the body becomes "symptomatic" and the patient describes physical constraints including pain and some degree of loss of function. That state seemed to be the situation that brought the patients into the therapy. 3) A third stage labeled as "cultivated immediacy" was described by practitioners and patients as well: it is experienced as a new relationship to the body characterized by acceptance, immediacy and the body experienced without objectification. 4) In the fourth state labeled "the subjective body" the body is experienced as a source of learning and meaning, by practitioners described as endowed with "intelligence" and an "innate tendency towards embodiment". In this state the body a) is no longer just the means by which the self carries out its projects or b) the source of constraints and limits to the self's goals, but rather an integral and equal part of the self and the locus of consciousness and subjectivity with its own perspective[55].

This dialectic of body and self formulated by phenomenological philosophers has been expanded to a body-self-environment "trialectic" by the practitioner and patient focus groups incorporating the person as embedded and active in a cultural environment and society[55]. Our participants clearly confirmed this view.

The findings of our focus groups with body-awareness practitioners and patients practicing a variety of these approaches are consistent with prior published qualitative data from research of specific mind-body approaches that are represented in our focus group or comparable to those involved. Qualitative data have been published for a number of body awareness-enhancing approaches including body awareness therapy as a form of physical therapy[19,28,58,59], qigong and body scan[60], massage[61], breath therapy[18], body-oriented psychotherapy[4], and Rosen Method Bodywork[62]. The data were collected in patients with chronic musculoskeletal pain conditions[18,19,28,59,62], cancer[58,61], sexual abuse PTSD[4] and by interviewing a qigong master[60]. Each of these studies confirms several of the points that emerged in our study. Taken together, these qualitative studies represent a growing body of evidence that body awareness-enhancing therapies may provide psychological and pain-related benefits for patients suffering from a variety of conditions.

Not all body-awareness based practices were represented in our focus groups, so it is possible that there are alternative perspectives on body awareness. Likewise, individual practitioners and patients shared their personal beliefs and experiences relative to their practices and these may or may not be generalizable. However, the consistency across practice disciplines and patient experiences suggests that the emergent themes reflect common theory, practice and experience perspectives on body awareness among those engaged in mind-body practices focused on body awareness.

In summary, this qualitative study examined the common ground of body awareness-enhancing mind-body therapies and adds additional evidence to the referenced qualitative studies of individual approaches. In addition, these findings suggest that we need to broaden the biomedical paradigm to consider and include a developmental model of embodiment in order to better understand how body awareness-enhancing therapies work[52,63]. This paradigmatic model has been proposed for several decades in philosophical and nursing literature[52,63]. It overcomes the mind-body split, as it still persists in the biomedical model and integrates the phenomenology of complex mind-body interactions, as they are experienced by practitioners and patients in body awareness-enhancing therapies.

## Competing interests

The authors declare that they have no competing interests.

## Authors' contributions

WM conceived of the study, obtained funding, led its design, coordination, and manuscript preparation and participated in the analyses; JW led the qualitative analyses and participated in manuscript preparation; JD, CP, CK, TS and AS participated in study conception, design and manuscript preparation; VG participated in study coordination, analyses and manuscript preparation; All authors read and approved the final manuscript.
